# Dietary acrylamide exposure in male F344 rats: Dataset of systemic oxidative stress and inflammation markers

**DOI:** 10.1016/j.dib.2016.02.024

**Published:** 2016-02-14

**Authors:** Xiaolei Jin, Melanie Coughlan, Jennifer Roberts, Rekha Mehta, Jayadev Raju

**Affiliations:** Regulatory Toxicology Research Division, Bureau of Chemical Safety, Food Directorate, Health Products and Food Branch, Health Canada, Ottawa, Ontario, Canada

**Keywords:** Acrylamide, Food safety, Systemic toxicity, Oxidative stress, Inflammation, 8-Hydroxydeoxyguanosine, Isoprostane, Total antioxidant capacity, Paraoxonase 1, C-reactive protein, Homocysteine, Oxidized low-density lipoprotein, Intercellular adhesion molecule-1, Thromboxin 2, Nε-(carboxymethyl)lysine

## Abstract

We previously reported that dietary acrylamide, at doses (10 and 50 mg/kg diet) known to cause rodent tumors, lowered serum total high density lipoprotein and total testosterone, increased serum lipase, and lowered lymphocytes levels together with other hematological parameters in male F344 rats exposed for 10 weeks (doi: 10.1016/j.etap.2014.11.009 [1]). Here we present data related to the role of food-borne acrylamide exposure (at 0, 5, 10 and 50 mg/kg diet) in the presence of low (7% wt/wt) or high (23.9% wt/wt) dietary fat on serum and urinary markers of oxidative stress and inflammation in F344 rats. Briefly, urine and serum samples were collected from the experimental animals a day prior to or at the time of necropsy, respectively and processed for enzyme-linked immunosorbent assay estimations of biochemical markers. Urine samples were analyzed for 8-hydroxydeoxyguanosine and isoprostane, and serum samples for total antioxidant capacity, paraoxonase 1 activity, c-reactive protein, homocysteine, oxidized low-density lipoprotein, intercellular adhesion molecule-1, thromboxin 2, and Nε-(carboxymethyl)lysine.

**Specifications Table**

**Table:** 

Subject area	*Toxicology*
More specific subject area	*Food toxicology, systemic oxidative stress and inflammation*
Type of data	*Figures, Table.*
How data was acquired	*All data was acquired using the POLARstar Omega multi-mode microplate reader(BMG LABTECH Inc., Cary, NC, USA)*
Data format	*Analyzed data*
Experimental factors	*Urine and serum from rats exposed to dietary acrylamide were processed and individual enzyme-linked immunosorbent assays (ELISAs) were performed to investigate markers of oxidative stress and inflammation.*
Experimental features	*Male F344 rats were fed an AIN−93 G basal diet containing low fat (7% wt/wt) or high fat (23.9% wt/wt) and 0, 5, 10, or 50 mg/kg diet acrylamide for 8 weeks. Rats were placed in metabolic cages 24 h before necropsy for urine sample collection. Blood samples were collected from abdominal aorta at necropsy*
Data source location	*Ottawa, Ontario, Canada*
Data accessibility	*Data is with this article*

## Value of the data

• We explored the role of chronic exposure to food-borne acrylamide in modulating markers of systemic oxidative stress and inflammation in rats under low and high fat diet conditions.
• This systemic biochemical data will support previous findings of acrylamide exposure at doses known to cause rodent tumors.
• Our data will be beneficial in updating the existing toxicity information available on food-borne acrylamide for regulatory purposes.

## 1 Data

**Fig. 1:**
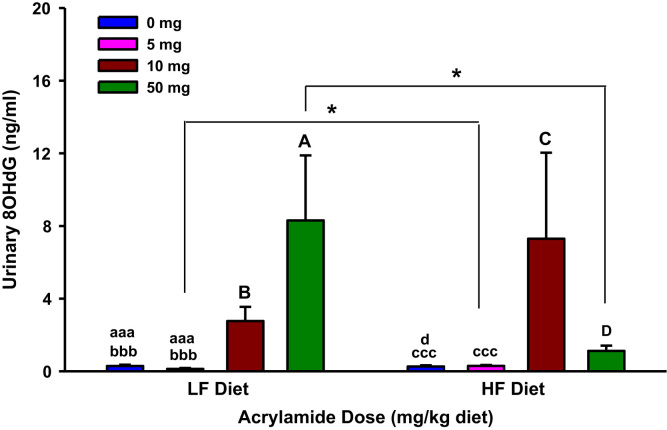
Urinary 8-hydroxydeoxyguanosine (8OHdG) levels in rats fed low fat (LF) or high fat (HF) diet and treated with acrylamide at 0, 5, 10 or 50 mg/kg diet, *n*=8/group. The histograms represent mean values±SEM. “aaa”, “bbb”, and “ccc” are significantly different from “A”, “B”, and “C” respectively at *p*<0.001. ”d” is significantly different from “D” at *p*<0.05. “*” indicates significant difference at *p*<0.05.

**Fig. 2:**
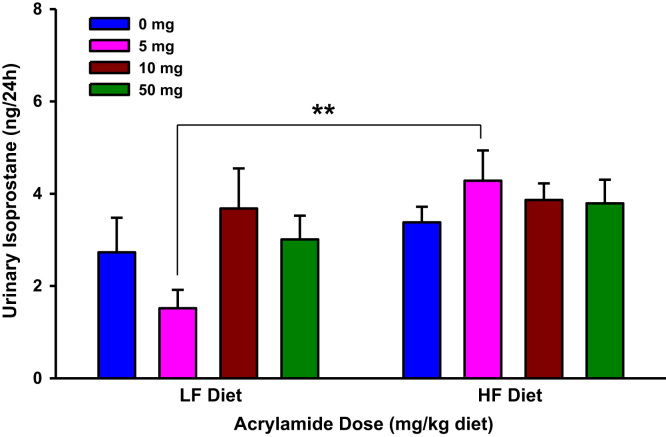
Urinary isoprostane levels in rats fed low fat (LF) or high fat (HF) diet and treated with acrylamide at 0, 5, 10 or 50 mg/kg diet, *n*=8/group. The histograms represent mean values±SEM. “*” indicates significant difference at *p*<0.05.

**Fig. 3:**
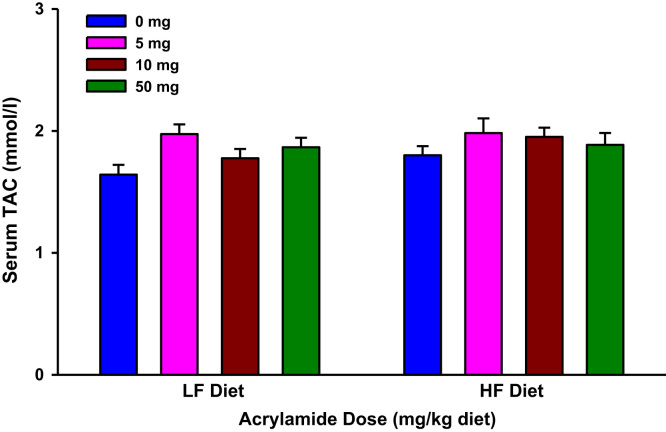
Serum total antioxidant capacity (TAC) levels in rats fed low fat (LF) or high fat (HF) diet and treated with acrylamide at 0, 5, 10 or 50 mg/kg diet, *n*=8/group. The histograms represent mean values±SEM.

**Fig. 4:**
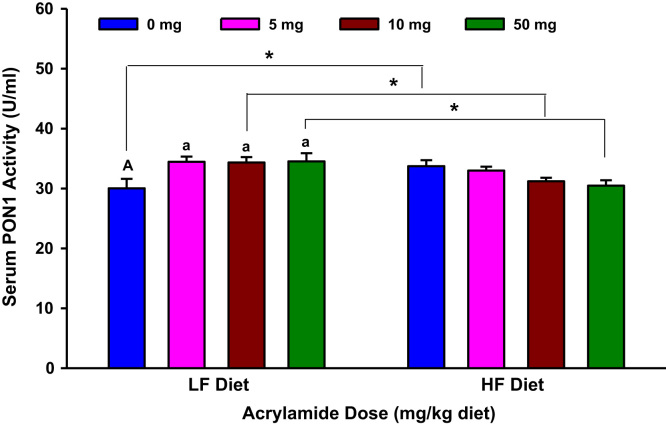
Serum paraoxonase 1 (PON1) activity in rats fed low fat (LF) or high fat (HF) diet and treated with acrylamide at 0, 5, 10 or 50 mg/kg diet, *n*=8/group. The histograms represent mean values±SEM. “a” is significantly different from “A” at *p*<0.05. “*” indicates significant difference at *p*<0.05.

**Fig. 5:**
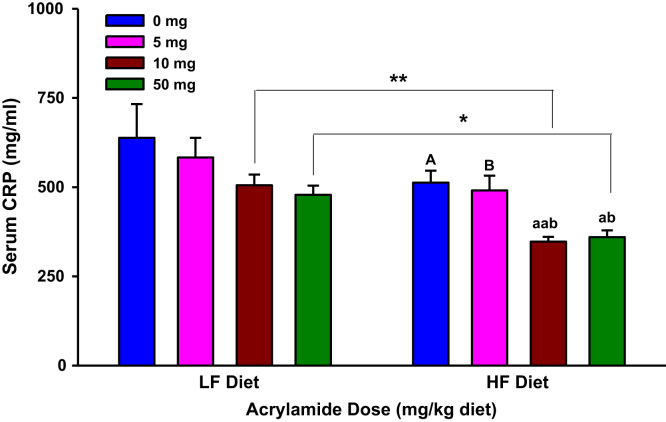
Serum c-reactive protein (CRP) levels in rats fed low fat (LF) or high fat (HF) diet and treated with acrylamide at 0, 5, 10 or 50 mg/kg diet, *n*=8/group. The histograms represent mean values±SEM. “a” and “b” are significantly different from “A” and “B” at *p*<0.05, respectively. “aa” is significantly different from “A” at *p*<0.01. “*” and “**” indicate significant difference at *p*<0.05 and *p*<0.01, respectively.

**Fig. 6:**
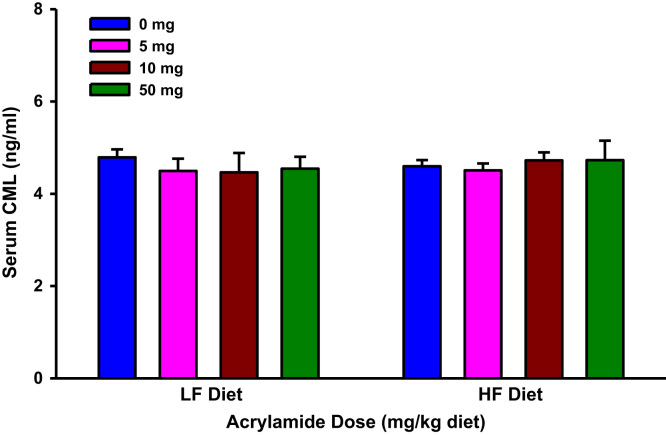
Serum homocysteine level in rats fed low fat (LF) or high fat (HF) diet and treated with acrylamide at 0, 5, 10 or 50 mg/kg diet, *n*=8/group. The histograms represent mean values±SEM. “aaa” is significantly different from “A” at *p*<0.001.

**Fig. 7:**
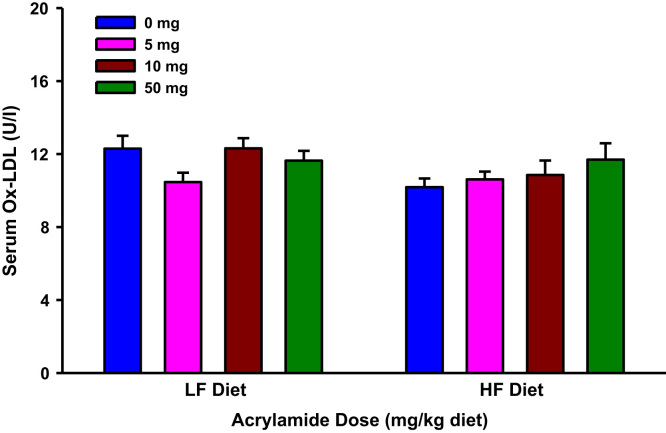
Serum oxidized low-density lipoprotein (Ox-LDL) levels in rats fed low fat (LF) or high fat (HF) diet and treated with acrylamide at 0, 5, 10 or 50 mg/kg diet), *n*=8/group. The histograms represent mean values±SEM.

**Fig. 8:**
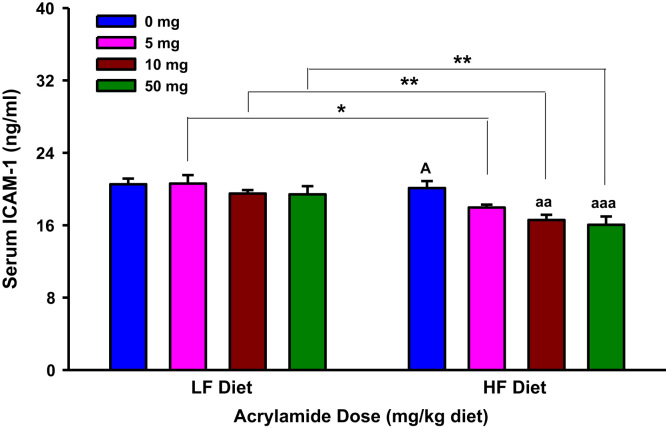
Serum intercellular adhesion molecule-1 (ICAM-1) levels in rats fed low fat (LF) or high fat (HF) diet and treated with acrylamide at 0, 5, 10 or 50 mg/kg diet, *n*=8/group. The histograms represent mean values±SEM. “aaa” is significantly different from “A” at p<0.001. “*” and “**” indicate significant difference at *p*<0.05 and *p*<0.01, respectively.

**Fig. 9:**
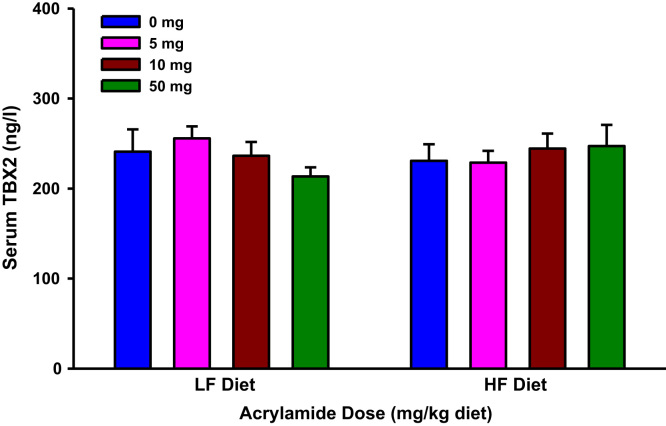
Serum thromboxin 2 (TBX2) levels in rats fed low fat (LF) or high fat (HF) diet and treated with acrylamide at 0, 5, 10 or 50 mg/kg diet, *n*=8/group. The histograms represent mean values±SEM.

**Fig. 10:**
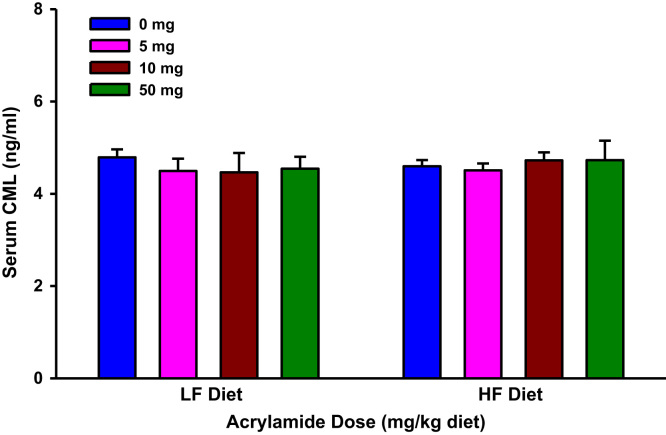
Serum Nε-(carboxymethyl)lysine (CML) levels in rats fed low fat (LF) or high fat (HF) diet and treated with acrylamide at 0, 5, 10 or 50 mg/kg diet. The histograms represent mean values±SEM, *n*=8/group.

The present dataset includes results of biochemical estimations that determined markers of systemic oxidative stress and inflammation in urine and serum samples of F344 rats exposed to dietary acrylamide (Fig. 1, Fig. 2, Fig. 3, Fig. 4, Fig. 5, Fig. 6, Fig. 7, Fig. 8, Fig. 9, Fig. 10; Table 2). These results of individual biochemical markers are to be interpreted with the clinical biochemistry, hematology and pathology data previously reported [1].

## 2 Experimental design, materials and methods

### 2.1 Animals, care and diets

The experimental protocol involving animals was reviewed and approved by the Health Canada Ottawa Animal Care Committee prior to the commencement of the study. Animals were cared for according to the guidelines of the Canadian Council on Animal Care. Six-week-old male F344 rats were procured from Charles River Laboratories Canada (St. Constant, Quebec, Canada) and were pair-housed in laboratory conditions with a 12 h light/12 h dark cycle. Temperature and relative humidity were controlled at 22 °C and 55%, respectively. All animals were acclimatized to the above conditions for 1 week until initiation of the experiment. The rats had free access to either lab chow (during the acclimatization phase) or experimental diets and drinking water *ad libitum*. The experimental diets were isocaloric and based on the AIN-93G rodent semisynthetic diet formula [2], but containing corn oil instead of soy oil as published earlier [3]. Fat level in the diet was maintained at either low (7%, wt/wt) or high (23.9%, wt/wt). Diets were obtained from Research Diets, Inc. (New Brunswick, NJ, USA) in the form of powder. Acrylamide was mixed with the diets at the required dose using a Hobart mixer, and then made into pellets using a pelleting press. Diets were never exposed to high temperature during processing and were stored in the dark at 4 °C until use. Rats were monitored every day and their body weights and food consumption were recorded twice a week; diets were replenished weekly.

### 2.2 Experimental design

After the acclimatization phase, animals (*n*=64) were randomized (2×4 factorial) into eight dietary groups (*n*=8 rats/group) to receive low or high fat diets without or with acrylamide (0, 5, 10 or 50 mg/kg diet). All animals remained on the experimental diets for a total of 10 weeks. A day before euthanasia, animals (not fasted) were placed in metabolic cages overnight, after which urine was collected (on ice) and urine volume was recorded for assay dilutions and calculations. Following urine collection, all rats were killed by exsanguination under isoflurane anesthesia, and blood was drawn from the abdominal aorta into BD Vacutainer SST™ blood collection tubes (Becton-Dickinson, Franklin Lakes, NJ, USA). Urine was centrifuged at 4000×*g* and serum was separated by centrifugation at 700×*g*, and in both cases the supernatant was collected, aliquots prepared, and stored at −80 °C until analysis.

### 2.3 Enzyme-linked immunosorbent assay (ELISA)

Urine samples were analyzed for 8-hydroxydeoxyguanosine and isoprostane, and serum samples for total antioxidant capacity, paraoxonase 1 activity, c-reactive protein, homocysteine, oxidized low-density lipoproetein, intercellular adhesion molecule-1, thromboxin 2, and Nε-(carboxymethyl)lysine carried out by the ELISA method using commercial kits according to the manufacturer׳s instructions. Details of the kits and test sample dilutions of individual assays are given in Table 1.

### 2.4 Statistical analysis

Data was analyzed performed using SigmaPlot 11.0. Statistical comparisons were performed using ANOVA with Tukey׳s post hoc test. For all tests, *p*<0.05 was considered as statistically significant.

## Figures and Tables

**Table 1:** Manufacturer details of commercial kits used and test sample dilution factor for individual assays.

Assay name	Catalog number	Manufacturer (City, Country)	Dilution of urine/blood

8-hydroxydeoxyguanosine	KOG-200 S/E	JaICA Shizuoka, Japan	1×urine
Isoprostane	EA85	Oxford Biomedical Research, Rochester Hills, MI, USA	10×urine
Total antioxidant capacity	NX 2332	Randox Laboratiories, Antrim, UK	1×serum
Paraoxonase 1	E33702	Thermo Fisher Scientific Inc. Waltham, MA, USA	170×serum
c-Reactive protein (CRP)	41-CRPRT-E01	Alpco Salem, NH, USA	12,000×serum
Homocysteine (HCy)	194-5361	Bio-Rad Laboratories, Inc. Hercules, CA, USA	1×serum
Oxidized LDL	10-1158-01	Mercodia AB Uppsala, Sweden	21×serum
Intercellular adhesion molecule-1	RIC100	R&D Systems Minneapolis, MN, USA	51×serum
Thromboxin 2	900-002	Assay Designs, Inc., Ann Arbor, MI, USA	400×serum
Nε-(carboxymethyl)lysine	CY-8066	CycLex Co., Ltd. Nagano-shi. Japan	6×serum

**Table 2:** Spearman correlation (coefficient) between acrylamide dose and oxidative stress and inflammatory markers.

Marker	8OHdG	Isoprostane	TAC	PON1	CRP	HCy	Ox-LDL	ICAM-1	TBX2	CML
Low fat diet										
Acrylamide	0.681***	NS	NS	0.418*	NS	−0.563**	NS	NS	NS	NS
8OHdG	NS	0.461*	NS	NS	NS	NS	NS	NS	NS	NS
Isoprostane	NS	NS	NS	0.519*	NS	NS	NS	NS	NS	NS
TAC	NS	NS	NS	NS	NS	NS	NS	NS	NS	NS
PON1	NS	NS	0.392*	NS	NS	NS	NS	−0.399*	NS	NS
CRP	NS	NS	NS	NS	NS	0.430*	NS	NS	NS	NS
Hcy	NS	NS	NS	NS	NS	NS	NS	0.443*	NS	NS
Ox-LDL	NS	NS	NS	NS	NS	NS	NS	0.500**	NS	NS
ICAM-1	NS	NS	NS	NS	NS	NS	NS	NS	NS	NS
TBX2	NS	NS	NS	NS	NS	NS	NS	NS	NS	NS
High fat diet										
Acrylamide	0.636***	NS	NS	−0.487**	−0.711***	NS	NS	−0.615***	NS	NS
8OHdG	NS	NS	NS	−0.363*	−0.769	−0.536**	NS	−0.646***	NS	NS
Isoprostane	NS	NS	NS	NS	NS	NS	0.509*	NS	NS	NS
TAC	NS	NS	NS	NS	NS	NS	NS	NS	NS	NS
PON1	NS	NS	NS	NS	0.408*	NS	NS	0.409*	NS	NS
CRP	NS	NS	NS	NS	NS	0.451**	NS	0.623***	NS	NS
Hcy	NS	NS	NS	NS	NS	NS	NS	0.380*	NS	NS
Ox-LDL	NS	NS	0.383*	NS	NS	NS	NS	NS	NS	NS
ICAM-1	NS	NS	NS	NS	NS	NS	NS	NS	NS	NS
TBX2	NS	NS	NS	NS	NS	NS	NS	NS	NS	NS

“*”,“**” and "***" indicate significant difference at *p*<0.05, *p*<0.01 and *p*<0.001, respectively.
